# Supplementary material for: Genomic Characterization of Salmonella enterica serovar Weltevreden Associated with Human Diarrhea
Source: Microbiol Spectr. 2023 Jan 18;11(1):e03542-22. doi: 10.1128/spectrum.03542-22 (PMC9927414; doi:10.1128/spectrum.03542-22)

Figure S1 A heatmap showing the predicted antimicrobial resistance genes (ARGs) in *S*. Weltevreden genomes.

Figure S2 A heatmap showing the predicted virulence factors encoding genes (VFGs) presence in *S*. Weltevreden genomes.

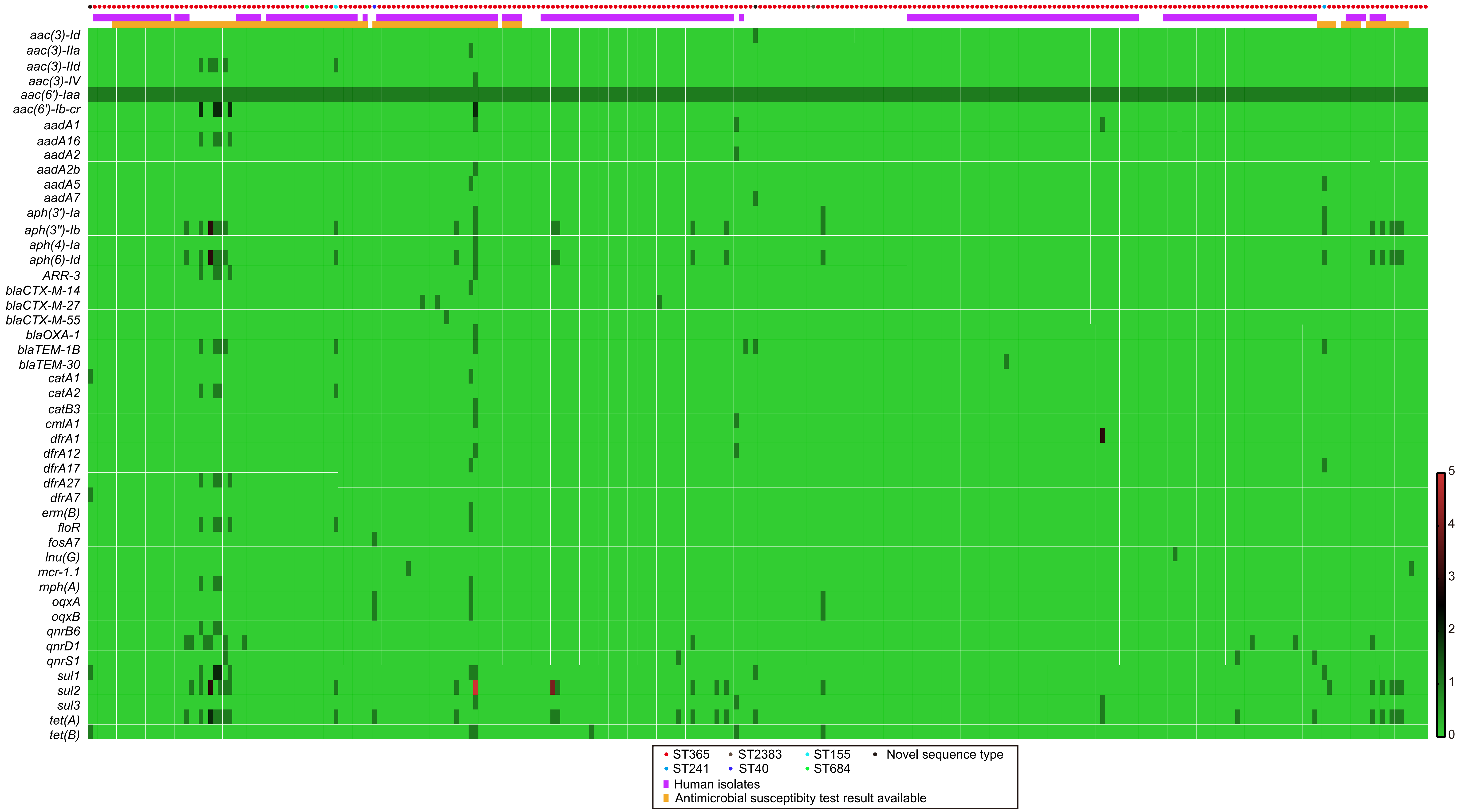
aac(3)-Id
aac(3)-IIa
aac(3)-IId
aac(3)-IV
aac(6')-Iaa
aac(6')-Ib-cr
aadA1
aadA16
aadA2
aadA2b
aadA5
aadA7
aph(3')-Ia
aph(3'')-Ib
aph(4)-Ia
aph(6)-Id
ARR-3
blaCTX-M-14
blaCTX-M-27
blaCTX-M-55
blaOXA-1
blaTEM-1B
blaTEM-30
catA1
catA2
catB3
cmlA1
dfrA1
dfrA12
dfrA17
dfrA27
dfrA7
erm(B)
floR
fosA7
lnu(G)
mcr-1.1
mph(A)
oqxA
oqxB
qnrB6
qnrD1
qnrS1
sul1
sul2
sul3
tet(A)
tet(B)
5
4
3
2
1
0
ST365
ST2383
ST155
Novel sequence type
ST241
ST40
ST684
Human isolates
Antimicrobial susceptibity test result available

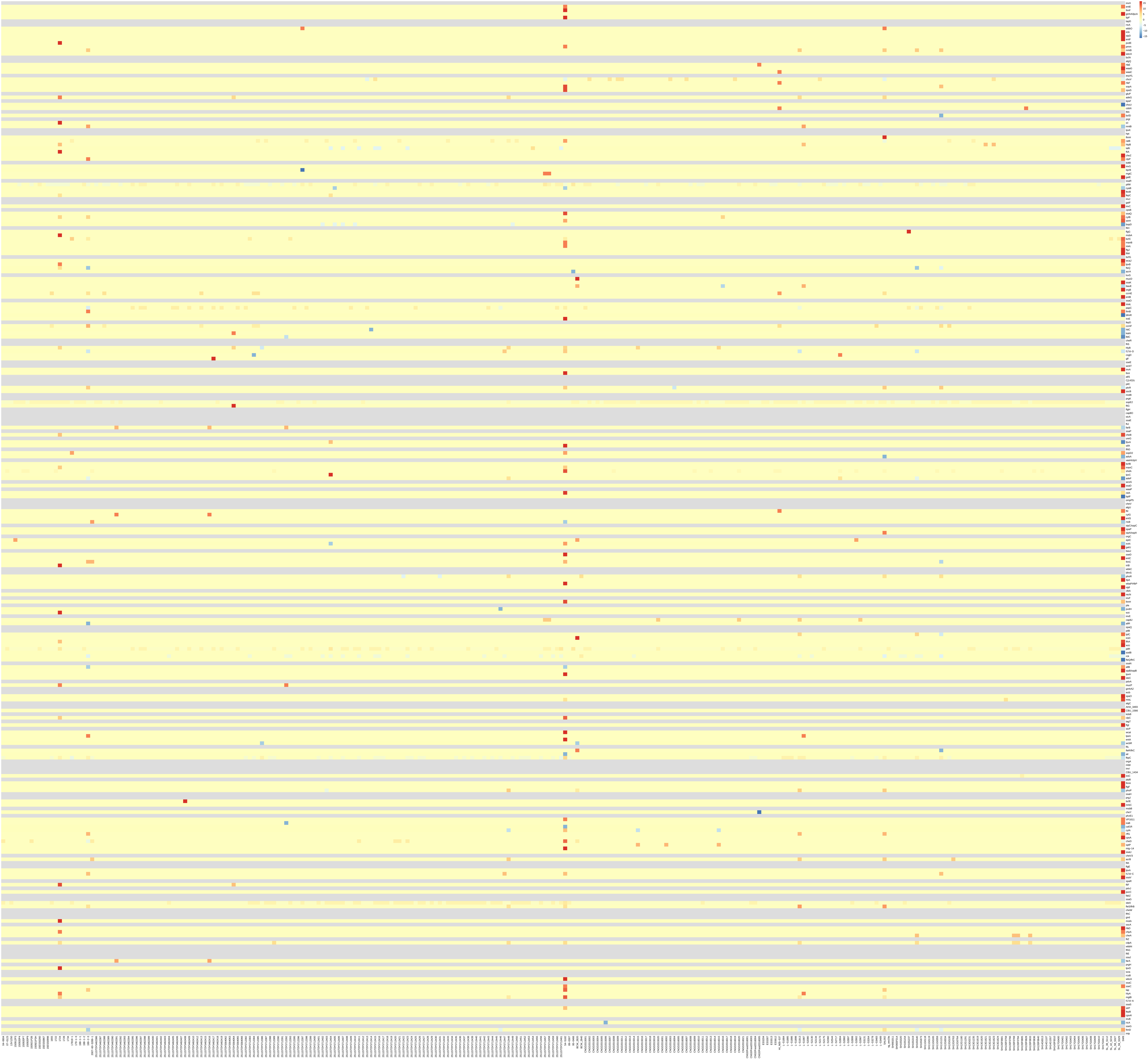

Supplement: Supplemental file 8 — Fig. S1 and S2. Download spectrum.03542-22-s0008.pdf, PDF file, 1.2 MB [file spectrum.03542-22-s0008.pdf]
